# The polymorphisms of IL-6/STAT3 signaling pathway may contribute to cutaneous T-cell lymphomas susceptibility

**DOI:** 10.1007/s00403-020-02062-5

**Published:** 2020-04-08

**Authors:** Berenika Olszewska, Jolanta Gleń, Monika Zabłotna, Roman J. Nowicki, Małgorzata Sokołowska-Wojdyło

**Affiliations:** Department of Dermatology, Venereology and Allergology, Medical University of Gdańsk, Mariana Smoluchowskiego Str 17, 80-214, Gdańsk, Poland

**Keywords:** Mycosis fungoides, Cutaneous lymphomas, IL-6, STAT3, Polymorphism

## Abstract

IL-6/STAT3 signaling pathway has been suggested to play a role in CTCL pathogenesis. Polymorphisms in STAT3 signaling pathway-related genes might be a risk factor for CTCL. However, the exact role of inherited gene polymorphisms of IL-6 and STAT3 in the pathogenesis of CTCL is still not fully understood. The aim was to examine whether IL-6 cytokine and polymorphisms of IL-6 and STAT3 gene are associated with CTCL susceptibility, stage of disease and pruritus intensity. We compared the IL-6 serum level and the frequency of selected single nucleotide polymorphisms of IL-6 and STAT3 in 106 CTCL and 198 control group using polymerase chain reaction with sequence-specific primers method and ELISA. We have found that serum IL-6 level in CTCL patients was significantly higher than in healthy controls (*p* < 0.05). We also demonstrated that two genotypes, CC of IL-6 and GG of STAT3, were overexpressed in CTCL patients compared to healthy controls, and that they increase the risk of malignancy development (OR = 1.8, *p* = 0.04 for IL-6 and OR 2.53, *p* = 0.0064 for STAT3). Moreover, the GG genotype of STAT3 polymorphism seems to be associated with lack of pruritus or mild pruritus in CTCL patients. Our results indicate that IL-6 is involved in pathogenesis of CTCL but not pruritus. Moreover, CC of IL-6 and GG genotype of STAT3 genes might be considered as the risk factor for development of CTCL.

## Introduction

Cutaneous T-cell lymphomas (CTCLs) comprise a clinically heterogeneous group of malignant limfoproliferative disorders. Most common subtypes are mycosis fungoides (MF) and Sezary syndrome (SS), an aggressive variant of CTCL [1]. CTCLs are characterized by cutaneous infiltrates of malignant CD4 + CD45RO + skin-homing T cells, eosinophilia and high levels of immunoglobulin E. MF display varying degrees of inflammation, early stage presents limited patches or plaques; however, they can eventually evolve into tumors or erythroderma [1, 2]. In minority of patients, the disease can further disseminate to the lymph nodes, blood and visceral organs [1, 2]. There are a few hypotheses of CTCL pathogenesis, including viral etiology or chronic antigen stimulation, e.g., exposure to UV radiation or bacterial superantigens such as *S. aureus* leading to development and clonal proliferation of malignant T cells [3–5]. However, results are contradictory and there is no evidence confirming that they are essential for the MF development. On the other hand, results of studies on genomic landscape of CTCL draw attention to genetic alterations in genes involved in signaling pathways or cellular processes [6–11]. Series of studies proved that the genomic basis of CTCL is rather heterogeneous and no pathognomonic mutations defining CTCL have been identified. However, there has been a great interest in genomic analysis of CTCL resulting in numerous studies identifying mutations affecting chromatin, immune surveillance, signaling pathways, cell cycle control, T-cell receptor signaling and T-cell differentiation [6–11]. The gene mutations provide novel targets in CTCL pathogenesis and thereby treatment opportunities. CTCL seems to be a complex series of interactions between different cells in skin microenvironment. The pathogenesis remains poorly elucidated; however, the inflammatory microenvironment of the skin appears to be of great importance in CTCL development and progression. CTCL progression is accompanied by the transition from type-1 T-helper cells (Th1) to type-2 T-helper cells (Th2) cytokine profile, therefore indicating the nature of immune response [12–16]. The predominance of Th2 cells over Th1 cells in inflammatory microenvironment is believed to be responsible for suppression of anti-tumor response, proliferation of malignant cells and escape from immunosurveillence [14–16]. As interleukin 6 plays a pivotal role during the transition from innate to acquired immunity, IL-6 has been shown to skew T-cell differentiation towards Th2 [17]; thus, it makes IL-6 a promising lead in the context of CTCL pathogenesis. IL-6 was initially considered to be a pro-inflammatory cytokine; however, we currently know that it also has anti-inflammatory activity [18]. Moreover, IL-6 is particularly interesting due to its involvement in the differentiation of immune cells toward Th2 phenotype by STAT3 signaling pathway. Janus kinase/signal transducer and activator of transcription (JAK/STAT) signaling pathway is responsible for cytokine signaling resulting in proliferation, migration and survival of cell. Deregulation of JAK/STAT pathway has been demonstrated in various cancers [19]. In particular, constitutive activation of STAT3 has been shown to play an essential role not only in acute myeloid leukemia, Hodgkin lymphoma, nasal-type natural killer cell lymphoma but also gastric cancer, ovarian cancer and breast cancer [20–23]. Moreover, also STAT3 polymorphism was associated with STAT3 expression and response to treatment [24]. JAK/STAT pathway deregulation seems to be crucial also for CTCL progression. Especially, deregulation of STAT3 signaling by inactivation of tumor suppressors (HNRNPK and SOCS1), which is a recently reported genetic alteration in CTCLs, might be responsible for disease development and progression [25]. Interestingly, STAT3 has been also suggested to be a malignancy factor in CTCL due to its potential to inhibit tumor cells apoptosis [26]. Constitutive activation of STAT3 has already been proven in tumor cells isolated from the patients with CTCL [27, 28]. However, the potential association between STAT3 polymorphism and CTCL has not been reported yet. Since CTCL is characterized by long-lasting course and rather rare aggressive progression, it is interesting whether the variability of IL-6 and STAT3 genes is associated with the course of disease.

The aim of the study was to assess the frequency of IL-6 -174 G/C gene polymorphisms in CTCL patients, relationship between IL-6 gene polymorphisms, IL-6 serum level and clinical values such as stage of the disease or pruritus intensity. Moreover, to extend the studies, we assessed the frequency and association of STAT3 polymorphisms and course of disease.

## Materials and methods

The study group included 106 patients with CTCL (100 MF, 6 SS) diagnosed and treated at the Department of Dermatology of the Medical University in Gdansk and a control non-CTCL group of 198 unrelated healthy individuals of the similar age and gender distribution, without personal or family history of chronic skin diseases and without personal history of malignancy. Patients with CTCL were diagnosed on the basis of clinical, histopathological and immunohistochemical findings, according to the European Organization of Research and Treatment of Cancer (EORTC) criteria [1]. Mycosis fungoides/SS patients were staged: IA (41 cases), IB (32 cases), IIA (1 case), IIB (13 cases), III (8 cases) and IV (5 cases) and SS (6 cases) according to the staging system proposed by the International Society of Cutaneous Lymphoma (ISCL) and the EORTC [29]. In the case of rs1800795 IL-6 gene polymorphism, 106 CTCL patients (men 68, women 38, mean age: 61.57 ± 15.37, range: 20–90) and 198 age- and sex-matched healthy individuals without history of pruritus, AD, CTCL or other immune diseases were examined. IL-6 serum level was evaluated in 67 CTCL patients and 48 healthy controls. The rs2293152 and rs4796793 STAT3 polymorphisms were performed in 104 CTCL and 198 healthy subjects. Moreover, pruritus intensity was evaluated according to visual analog scale (VAS)/numerical analog scale (NRS) and correlated with IL-6 serum level, IL-6 and STAT3 gene polymorphisms. The study was approved by the local research ethics committee of the Medical University of Gdansk.

### DNA extraction/genotyping and IL-6 serum level

Genomic DNA was isolated from all the blood samples with Blood Mini A&A Biotechnology (A&A Biotechnology, Gdansk, Poland) according to the instructions of the manufacturer. Analysis of the polymorphic variants IL-6 rs1800795 (-174 G/C) and in the STAT3 gene: rs2293152 and rs4796793 were analyzed with the use of polymerase chain reaction with sequence-specific primers (SSP-PCR). Serum IL-6 levels were measured with an enzyme-linked immunoabsorbent assay (ELISA) standard kit (Human IL-6 High Sensitivity ELISA kit, Diaclone SAS, France).

### Statistical analyses

Statistical calculations were made with the use of Statistica, version 12.0. (StatSoft, Inc. 2015). Analysis of qualitative features was made with the *χ*2 test in the Pearson method. Independent variables fulfilling the assumptions for parametric tests were analyzed with the Student’s *t* test. Independent variables that did not meet the parametric test assumptions were analyzed with non-parametric tests (ANOVA equivalents): *U* Mann–Whitney test (comparison of two tests) or Kruskal–Wallis test (comparison of many samples). Odds ratios (ORs) with 95% confidence intervals were determined by logistic regression. In all tests, *p* < 0.05 was considered a significant level of statistical significance.

## Results

### IL-6 serum level

**Fig. 1:**
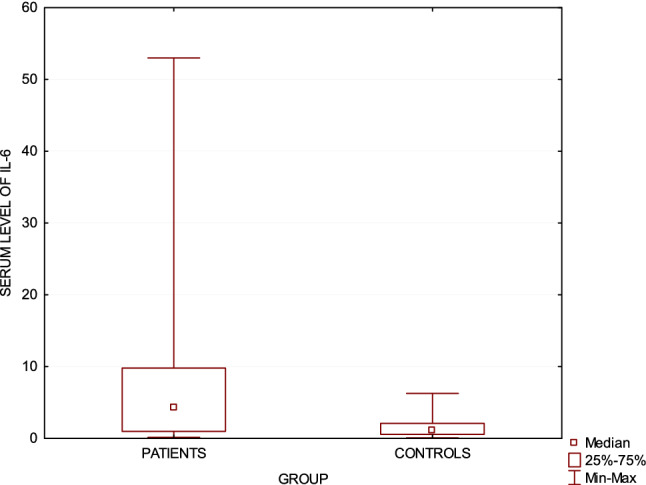
Serum IL-6 level in cutaneous T-cell lymphomas patients compared to healthy controls (*p* < 0.05)

The median and mean ± SD serum IL-6 levels in CTCL patients were significantly higher than in healthy controls (6.85 ± 8.78 pg/ml; 4.31 pg/ml; range: 0.14–53.0 compared to 1.4 ± 1.28 pg/ml; 1.05 pg/ml; range: 0.08–6.27; *p* < 0.05) (Fig. 1). No statistically significant differences were found in serum IL-6 level between early and advanced stages of the disease (*p* = 0.94). There were no statistically significant differences in serum IL-6 level between pruritic and non-pruritic CTCL patients (VAS *p* = 0.71; NRS *p* = 0.84) No positive correlation was found between serum IL-6 levels and severity of pruritus in CTCL (VAS *p* = 0.68; NRS *p* = 0.58).

### The IL-6 gene polymorphisms

**Fig. 2:**
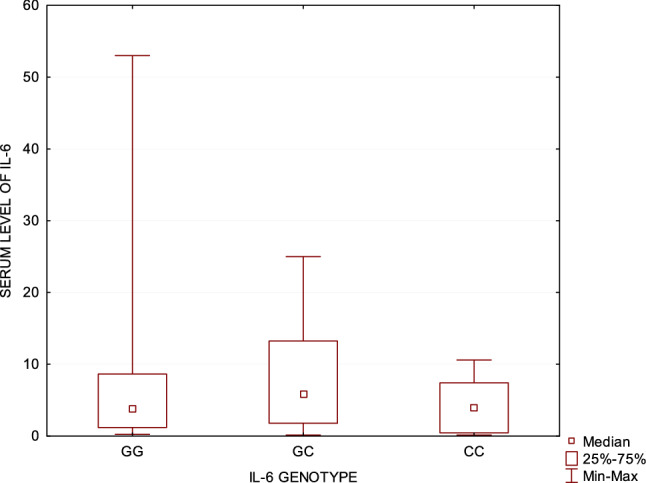
IL-6 genotype in relation to IL-6 serum level in cutaneous T-cell lymphomas patients

The IL-6 genotype distribution is displayed in Table 1. The frequencies of the GG, GC and CC in IL-6 genotype in CTCL were 51.89%, 22.64% and 25.47%, respectively. We found a significant difference in IL-6 genotype distribution between patients and control group (*p* = 0.01). The homozygote CC genotype of IL-6 gene polymorphism occurred more frequently in CTCL patients than healthy controls (*p* = 0.037). Presence of IL-6 CC genotype significantly increases the risk of CTCL (OR = 1.8, 95% CI 1.03–3.29, *p* = 0.04). The distinction between the genotype frequency of IL-6 gene polymorphism in different stages of CTCL and pruritus intensity was also examined. The genotype CC of IL-6 gene was found to be statistically more frequent in early than advanced stage of CTCL (*p* = 0.04). Presence of IL6 CC genotype significantly increased the risk of early stage of CTCL (OR = 3.22, 95% CI 1.01–10.25, *p* = 0.048). There were no significant differences in IL-6 genotype distribution between pruritic and non-pruritic CTCL patients. However, the genotype CC of IL-6 gene was statistically more frequent in CTCL patients accompanied by severe pruritus (*p* = 0.04 VAS scale). Presence of IL-6 CC genotype significantly increases the risk of severe pruritus (OR = 2.63, 95% CI 1.05–6.59, *p* = 0.04). The homozygote GG was statistically more frequent in CTCL patients affected by mild pruritus (*p* = 0.03 VAS scale). Presence of IL6 GG genotype significantly increased the risk of mild pruritus (OR = 0.41, CI 0.18–0.92, *p* = 0.03). The CTCL group with genotype GG of IL-6 gene presented significantly lower mean pruritus intensity compared to other genotypes (NRS *p* = 0.02/VAS *p* = 0.05). No significant differences in IL-6 serum level between different − 174 G/C polymorphisms of IL-6 gene were observed (Fig. 2).

**Table 1:** Frequency of interleukin-6 gene polymorphisms and association with itch intensity and stage of disease in CTCL patients and healthy controls

Polymorphism rs1800795 – 174 G/C of interleukin-6		
	CTCL	Control group
Genotypes	*n* = 106	*n* = 198
GG	55 (51.89%)	93 (46.97%)
GC	24 (22.64%)	74 (37.37%)
CC	**27 (25.47%)**	31 (15.66%)
	CTCL mild pruritus (VAS)	CTCL severe pruritus (VAS)
Genotypes	*n* = 56	*n* = 44
GG	**34 (60.71%)**	17 (38.64%)
GC	12 (21.43%)	11 (25.0%)
CC	10 (17.86%)	**16 (36.36%)**
	Early stage CTCL	Advanced stage CTCL
Genotypes	*n* = 73	*n* = 32
GG + GC	50 (68.49%)	28 (87.5%)
CC	**23 (31.51%)**	4 (12.5%)

Values in bold indicate statistical significance (*p* < 0.05)

### The STAT3 gene polymorphisms

No significant difference in rs2293152 genotype distribution was found between CTCL group and control group. The allelic frequency of rs2293152 STAT3 polymorphism did not differ between both groups either. However, the study revealed that rs4796793 GG genotype is statistically more frequent in CTCL patients than control group (*p* = 0.005) (Table 2.). The presence of rs4796793 GG genotype significantly raised the risk of the disease (OR 2.52, 95% CI 1.3–4.93, *p* = 0.006). The rs4796793 G allele was more frequent in CTCL patients than control group (*p* = 0.005). Presence of STAT3 rs4796793 G allele increases the risk of CTCL (OR = 1.65, 95% CI 1.16–2.34, *p* = 0.005). No statistically significant differences were found in the genotype frequencies for rs2293152 and rs4796793 STAT3 gene polymorphism in the subgroups of early- and advanced-stage CTCL patients. STAT3 gene polymorphisms do not represent a risk factor for developing a certain stage of disease. There were no significant differences in rs2293152 IL-6 genotype distribution between pruritic and non-pruritic CTCL patients. Interestingly, the GG genotype of rs4796793 STAT3 polymorphism was significantly more frequent in CTCL patients without pruritus (*p* = 0.01). The presence of STAT3 rs4796793 GG genotype was strongly associated with lack of pruritus in CTCL (OR = 3.65, 95% CI 1.31–10.19, *p* = 0.014). Moreover, presence of GG genotype of rs4796793 was more frequent in CTCL subgroup with mild pruritus (*p* = 0.04). We observed a tendency that the presence of rs4796793 GG genotype increases the probability of mild rather than strong itch occurrence in CTCL (OR = 3.0, 95% CI 0.99–9.06, *p* = 0.05). No significant differences in IL-6 serum level in CTCL patients and healthy controls, between STAT3 polymorphisms in CTCL patients were observed.

**Table 2:** Frequency of STAT3 gene polymorphisms and association with itch intensity in CTCL patients and healthy controls

Polymorphism rs4796793 of STAT3		
	CTCL	Control group
Genotypes	*n* = 104	*n* = 198
GG	**22 (21.15%)**	19 (9.6%)
GC	41 (39.42%)	79 (39.90%)
CC	41 (39.42%)	100 (50.51%)
	CTCL mild pruritus (VAS)	CTCL severe pruritus (VAS)
Genotypes	*n* = 54	*n* = 44
GG	**15 (27.78%)**	5 (11.36%)
GC	18 (33.33%)	22 (50.0%)
CC	21 (38.64%)	17 (38.64%)

Values in bold indicate statistical significance (*p* < 0.05)

## Discussion

Deregulation of IL-6 expression and JAK/STAT pathway most likely contribute to the development and progression of CTCL; however, the exact mechanism is still not fully understood. IL-6 has been reported to be elevated in serum of MF patients [30, 31]. Moreover, serum levels of IL-6 were demonstrated to correlate with tumor burden in Sézary syndrome and with clinical stage in non-leukemic CTCL [31]. The IL-6 -174G/C polymorphism that we have studied in CTCL has already been proved to be related to inflammatory and autoimmune diseases [32–34]. Data including IL-6 polymorphisms in CTCL are rather limited and conflicting; however, the heterozygote variant of − 596 A/G promoter IL-6 polymorphism has been suggested a genotype marker for CTCL [35]. On the other hand, deregulation of STAT signaling plays a key role in pathogenesis of CTCL. STAT3 most likely takes part in CTCL progression, as its constitutive expression in advanced stages of this cancer has been observed [26, 36, 37]. In the resent study, Perez and coworkers provided the evidence that STAT3 is activated in advanced- rather than early-stage CTCL cases and it is associated with large-cell transformation that seems to be a predictor of progression [37]. Moreover, Lindahl and coworkers reported inhibition of tumor cells and disease activity in patients with advanced-stage CTCL after antibiotic treatment [38]. A decrease in STAT3 expression accompanied by the clinical improvement was also observed, which supports the hypothesis that enhancing STAT3 activation and, therefore, disease progression might depend on *S. aureus* and its toxins [38]. Furthermore, activation of STAT3 seems to promote survival and resistance to apoptosis [28], it upregulates oncogenic miR-155 microRNA that downregulates STAT4 and, therefore, promotes Th2 phenotype [39]. STAT3 upregulation in CTCL is proved, but the relationship between STAT3 genetic variants and CTCL has not been studied yet.

Polymorphisms of various cytokines in CTCL and their association with disease development have already been studied [40–43]; however, to the best of our knowledge, there is no publication analyzing IL-6 serum level together with IL-6 and STAT3 gene polymorphisms in CTCL. The aim of the study was to assess the frequency of IL-6 gene polymorphisms in CTCL patients, relationship between IL-6/STAT3 gene polymorphisms and IL-6 serum level and clinical values such as stage of the disease and pruritus.

This study found significantly increased serum level of IL-6 in CTCL patients, that is in line with previous studies [30, 31, 44]. In contrast to Hassel et al. study, we did not observe correlation between CTCL clinical stage and IL-6 serum level [31]. Overexpression of IL-6 in CTCL compared to healthy controls proves its involvement in the CTCL pathogenesis.

Moreover, overexpression of IL-6 results in over-activated receptor signaling that contributes to the constitutive activation of STAT3 that has been observed in advanced stages of CTCL [26]. It seems that IL-6 might contribute to CTCL progression by over-activated STAT3 signaling but certainly IL-6 plays a significant role in inflammation process in CTCL [17]. We did not find correlation between IL-6 serum level and pruritus intensity, indicating lack of pruritogenic potential of IL-6. Interestingly, at the same time, we also observed that the genotype − 174 CC of IL-6 gene was statistically more frequent in severe pruritus, while GG genotype in mild pruritus.

Moreover, we have demonstrated significant differences in genotype distribution of − 174 G/C IL-6 polymorphism between patients and control groups. Our findings are contrary to Vasku et al. study and Hodak et al. study that did not find any differences in that genotype distribution and frequency between both groups [35, 45]. However, a different heterozygote variant of promoter IL-6 polymorphism (− 596 A/G) was indicated to be a potential genotype marker due to threefold odds ratio for CTCL [35]; in our study, – 174 CC IL-6 genotype showed nearly twofold odds ratio for CTCL. We believe that those results should be interpreted with caution as the reports including the same − 174 G/C IL-6 polymorphism are incompatible and therefore, further studies should be performed [35, 45]. Nonetheless, we can conclude that patients homozygous for C allele are more susceptible to CTCL than those homozygous and/or heterozygous for G allele. It has been reported that the C allele of − 174 G/C IL-6 gene polymorphism was associated with significantly lower IL-6 level in control group than juvenile arthritis [46]. However, in case of CTCL, we did not observe any relationships either between − 174 G/C IL-6 polymorphisms or STAT3 polymorphisms, and IL-6 serum level.

The association of STAT3 polymorphism rs4796793 with cancer susceptibility has been already demonstrated in lung cancer [47], breast cancer [48], hepatocellular carcinoma [49] and cervical cancer [50]. However, its association with CTCL has not been studied yet. We found that STAT3 rs4769793 G allele carriers had an increased risk of CTCL (one-and-a-half-fold odds ratio); while, patients with homozygote GG genotype of rs4796793 STAT3 had even greater susceptibility to this disease (two-and-a-half-fold odds ratio). Our results indicate that the GG genotype of rs4796793 STAT3 polymorphism might be considered a risk factor for CTCL development and it might potentially contribute to disease progression. Further clinical observations would be of great value to assess whether CTCL disease in patients with the GG genotype of rs4796793 STAT3 are more prone to progress or rather remain stable. In the light of recent reports, it would be also worth to analyze whether STAT3 polymorphisms determine its susceptibility to *S. aureus* promotion and, therefore, the course of disease [38]. We also found that the presence of STAT3 rs4796793 GG genotype was strongly related to lack of pruritus or mild pruritus. It seems that GG genotype has a protective association when it comes to pruritus. However, there are several limitations of our study that should be considered. First, the number of cases analyzed in this study was relatively small. Second, there is a lack of functional analysis of the association between IL-6/ STAT3 polymorphisms and prognosis of CTCL. Therefore, further studies on different levels would be of great value to better understand the etiology of CTCL.

In summary, our investigations show that IL-6 is involved in CTCL pathogenesis. Moreover, our study found that − 174 CC of IL-6 gene and GG genotype of rs4796793 STAT3 are associated with CTCL in the Polish population and they might be considered potential candidates’ biomarkers for the prediction of susceptibility to CTCL.

## References

[1] Willemze R, Cerroni L, Kempf W (2019). The 2018 update of the WHO-EORTC classification for primary cutaneous lymphomas. Blood.

[2] Wilcox RA (2016). Cutaneous T-cell lymphoma: 2016 update on diagnosis, risk stratification, and management. Am J Hematol.

[3] Morales-Suárez-Varela MM, Olsen J, Johansen P (2006). Occupational sun exposure and mycosis fungoides: a European multicenter case-control study. J Occup Environ Med.

[4] Vonderheid EC, Bigler RD, Hou JS (2005). On the possible relationship between staphylococcal superantigens and increased Vbeta5.1 usage in cutaneous T- cell lymphoma. Br J Dermatol.

[5] Jackow CM, Cather JC, Hearne V (1997). Association of erythrodermic cutaneous T- cell lymphoma, superantigen-positive *Staphylococcus aureus*, and oligoclonal T-cell receptor V beta gene expansion. Blood.

[6] McGirt LY, Jia P, Baerenwald DA (2015). Whole-genome sequencing reveals oncogenic mutations in mycosis fungoides. Blood.

[7] Wang L, Ni X, Covington KR (2015). Genomic profiling of Sezary syndrome identifies alterations of key T cell signaling and differentiation genes. Nat Genet.

[8] Park J, Yang J, Wenzel AT (2017). Genomic analysis of 220 CTCLs identifies a novel recurrent gain-of-function alteration in RLTPR (p. Q575E). Blood.

[9] Litvinov IV, Tetzlaff MT, Thibault P (2017). Gene expression analysis in cutaneous T-cell lymphomas (CTCL) highlights disease heterogeneity and potential diagnostic and prognostic indicators. Oncoimmunology.

[10] Woollard WJ, Pullabhatla V, Lorenc A (2016). Candidate driver genes in Sezary syndrome: frequent perturbations of genes involved in genome maintenance and DNA repair. Blood.

[11] da Silva Almeida AC, Abate F, Khiabanian H (2015). The mutational landscape of cutaneous T cell lymphoma and Sezary syndrome. Nat Genet.

[12] Wong HK, Mishra A, Hake T (2011). Evolving Insights in the pathogenesis and therapy of cutaneous T-cell lymphoma (Mycosis Fungoides and Sezary Syndrome). Br J Haemat.

[13] Vowels BR, Lessin SR, Cassin M (1994). Th2 cytokine mRNA expression in skin in cutaneous T-cell lymphoma. J Invest Dermatol.

[14] Hoppe RT, Medeiros LJ, Warnke RA (1995). CD8-positive tumor-infiltrating lymphocytes influence the long-term survival of patients with mycosis fungoides. J Am Acad Dermatol.

[15] Guenova E, Watanabe R, Teague JE (2013). TH2 cytokines from malignant cells suppress TH1 responses and enforce a global TH2 bias in leukemic cutaneous T-cell lymphoma. Clin Cancer Res.

[16] Chong BF, Wilson AJ, Gibson HM (2008). Immune function abnormalities in peripheral blood mononuclear cell cytokine expression differentiates stages of cutaneous T-cell lymphoma/mycosis fungoides. Clin Cancer Res.

[17] Tanaka T, Narazaki M, Kishimoto T (2014). IL-6 in inflammation, immunity, and disease. Cold Spring Harb Perspect Biol.

[18] Wolf J, Rose-John S, Garbers C (2014). Interleukin-6 and its receptors: a highly regulated and dynamic system. Cytokine.

[19] Bromberg J (2001). Stat proteins and oncogenesis. J Clin Invest.

[20] Holtick U, Vockerodt M, Pinkert D (2005). STAT3 is essential for Hodgkin lymphoma cell proliferation and is a target of tyrphostin AG17 which confers sensitization for apoptosis. Leukemia.

[21] Benekli M, Xia Z, Donohue KA (2002). Constitutive activity of signal transducer and activator of transcription 3 protein in acute myeloid leukemia blasts is associated with short disease-free survival. Blood.

[22] Coppo P, Gouilleux-Gruart V, Huang Y (2009). STAT3 transcription factor is constitutively activated and is oncogenic in nasal-type NK/T-cell lymphoma. Leukemia.

[23] Yu H, Lee H, Herrmann A (2014). Revisiting STAT3 signalling in cancer: new and unexpected biological functions. Nat Rev Cancer.

[24] Aggarwal BB, Kunnumakkara AB, Harikumar KB (2009). Signal transducer and activator of transcription-3, inflammation, and cancer: how intimate is the relationship?. Ann NY Acad Sci.

[25] Bastidas Torres AN, Cats D, Mei H (2018). Genomic analysis reveals recurrent deletion of JAK-STAT signaling inhibitors HNRNPK and SOCS1 in mycosis fungoides. Genes Chromosom Cancer.

[26] Sommer VH, Clemmensen OJ, Nielsen O (2004). In vivo activation of STAT3 in cutaneous T-cell lymphoma. Evidence for an antiapoptotic function of STAT3. Leukemia.

[27] Nielsen M, Kaltoft K, Nordahl M (1997). Constitutive activation of a slowly migrating isoform of Stat3 in mycosis fungoides: tyrphostin AG490 inhibits Stat3 activation and growth of mycosis fungoides tumor cell lines. Proc Natl Acad Sci USA.

[28] Nielsen M, Kaestel CG, Eriksen KW (1999). Inhibition of constitutively activated Stat3 correlates with altered Bcl-2/Bax expression and induction of apoptosis in mycosis fungoides tumor cells. Leukemia.

[29] Kim YH, Willemze R, Pimpinelli N (2007). TNM classification system for primary cutaneous lymphomas other than mycosis fungoides and Sezary syndrome: a proposal of the International Society for Cutaneous Lymphomas (ISCL) and the Cutaneous Lymphoma Task Force of the European Organization of Research and Treatment of Cancer (EORTC). Blood.

[30] Lawlor F, Smith NP, Camp RD (1990). Skin exudate levels of interleukin 6, interleukin 1 and other cytokines in my- cosis fungoides. Br J Dermatol.

[31] Hassel JC, Meier R, Joller-Jemelka H (2004). Serological immunomarkers in cutaneous T cell lymphoma. Dermatology.

[32] Schotte H, Schluter B, Rust S (2001). Interleukin-6 promoter polymorphism (– 174G/C) in Caucasian German patients with systemic lupus erythematosus. Rheumatol.

[33] Fishman D, Faulds G, Jeffery R (1998). The effect of novel polymor- phisms in the interleukin-6 (IL-6) gene on IL-6 transcrip- tion and plasma IL-6 levels, and an association with sys- temic-onset juvenile chronic arthritis. J Clin Invest.

[34] Pascual M, Nieto A, Mataran L (2000). IL-6 promoter polymorphisms in rheumatoid arthritis. Genes Immun.

[35] Vasku JA, Vasku A, Goldbergova M (2004). Heterozygote AG variant of− 596 A/G IL-6 gene polymorphism is a marker for cutaneous T-cell lymphoma (CTCL). Clin Immunol.

[36] Eriksen KW, Kaltoft K, Mikkelsen G (2001). Constitutive STAT3-activation in Sezary syndrome: tyrphostin AG490 inhibits STAT3-activation, interleukin-2 receptor expression and growth of leukemic Sezary cells. Leukemia.

[37] Pérez C, Mondéjar R, García-Díaz N (2020). Advanced-stage mycosis fungoides: role of the signal transducer and activator of transcription 3, nuclear factor-κB and nuclear factor of activated T cells pathways. Br J Dermatol.

[38] Lindahl LM, Willerslev-Olsen A, Gjerdrum LMR (2019). Antibiotics inhibit tumor and disease activity in cutaneous T-cell lymphoma. Blood.

[39] Van Der Fits L, Van Kester MS, Qin Y (2001). MicroRNA-21 expression in CD4+ T cells is regulated by STAT3 and is pathologically involved in Sezary syndrome. J Invest Dermatol.

[40] Vasku V, Bienertovau-Vasku J, Paavkova-Goldbergova M (2006). Association of polymorphic variants in endothelin-1 (EDN1) genes with the therapy of patients with cutaneous T-cell lymphomas. Cas Lek Cesk.

[41] Vasku A, Vasku JB, Necas M (2010). Matrix metalloprotein- ase-2 promoter genotype as a marker of cutaneous T-cell lymphoma early stage. J Biomed Biotechnol.

[42] Bellei B, Cota C, Amantea A (2006). Association of p53 Arg- 72Pro polymorphism and beta-catenin accumulation in my- cosis fungoides. Br J Dermatol.

[43] Nedoszytko B, Olszewska B, Roszkiewicz J (2016). The role of polymorphism of interleukin-2, -10, -13 and TNF-α genes in cutaneous T-cell lymphoma pathogenesis. Adv Dermatol Allergol. XXXIII.

[44] Kadin ME, Pavlov IY, Delgado JC (2012). High soluble CD30, CD25, and IL-6 may identify patients with worse survival in CD30+ cutaneous lymphomas and early mycosis fungoides. J Invest Dermatol.

[45] Hodak E, Akerman L, David M (2005). Cytokine gene polymorphisms in patch-stage mycosis fungoides. Acta Derm Vene- reol.

[46] Fishman D, Faulds G, Jeffery R (1998). The effect of novel polymorphisms in the interleukin-6 (IL-6) gene on IL-6 transcription and plasma IL-6 levels and an association with systemic-onset juvenile chronic arthritis. J Clin Invest.

[47] Gong WJ, Ma LY, Hu L (2019). STAT3 rs4796793 contributes to lung cancer risk and clinical outcomes of platinum-based chemotherapy. Int J Cin Oncol.

[48] Zhao L, Zhang Q, Luan X (2015). STAT3 and STAT5b polymorphism contributes to breast cancer risk and clinical outcomes. Int J Clin Exp Pathol.

[49] Xie J, Zhang Y, Zhang Q (2013). Interaction of signal transducer and activator of transcription 3 polymorphisms with hepatitis B virus mutations in hepatocellular carcinoma. Hepatology.

[50] Wang KN, Zhou B, Zhang J (2011). Association of signal transducer and activator of transcription 3 gene polymorphisms with cervical cancer in chinese women. DNA Cell Biol.

